# Cryptotanshinone hinders renal fibrosis and epithelial transdifferentiation in obstructive nephropathy by inhibiting TGF-β1/Smad3/integrin β1 signal

**DOI:** 10.18632/oncotarget.23803

**Published:** 2017-12-27

**Authors:** Wei Wang, Pang-Hu Zhou, Wei Hu, Chang-Geng Xu, Xiang-Jun Zhou, Chao-Zhao Liang, Jie Zhang

**Affiliations:** ^1^ Department of Urology, The First Affiliated Hospital of Anhui Medical University and Institute of Urology, Anhui Medical University, Hefei, Anhui, 232200, China; ^2^ Department of Urology, Renmin Hospital of Wuhan University, Wuhan, Hubei Province, 430060, China; ^3^ Department of Orthopedics, Renmin Hospital of Wuhan University, Wuhan, Hubei Province, 430060, China; ^4^ Department of Urology, The First Affiliated Hospital of Nan-Hua University, Henyang, Hunan, 421001, China; ^5^ Department of Urology, Wuhan Central Hospital, Wuhan, Hubei Province, 430014, China; ^6^ Department of Urology, Renmin Hospital of Wuhan University, Wuhan, Hubei Province, 430060, China; ^7^ Huangshi Central Hospital, Hubei Polytechnic University, Huangshi, Hubei Province, 435000, China

**Keywords:** cryptotanshinone, kidney fibrosis, epithelial-mesenchymal transition, Smad3, integrin β1

## Abstract

Recent studies have reported that CTS can alleviate cardiac fibrosis. However, the effects of CTS on kidney fibrosis and EMT are still unknown. This study explored whether CTS could attenuate tubulointerstitial fibrosis as well as EMT, and investigated the potential underlying mechanisms. In this study, an *in vivo* UUO mouse model and an *in vitro* TGF-β1 stimulated normal renal tubular kidney epithelial cell model were established. In UUO model, administration of 50 mg kg-1 day-1 CTS markedly decreased the occurrence of kidney injury and the accumulation of fibronectin and collagen-1. In addition, CTS reduced the expression level of α-SMA but retained E-cadherin in obstructed kidneys. *In vitro*, CTS suppressed the expression of fibronectin, collagen-1 and α-SMA but retained that of E-cadherin. Furthermore, CTS selectively abolished the activation of Smad3 and suppressed the nuclear translocation of Smad2, Smad3 and Smad4. CTS could block the promoter activity of integrin β1 induced by Smad3. Furthermore, CTS inhibited Smad3 binding to integrin β1 promoter sequences. These data suggest that CTS can ameliorate kidney fibrosis and EMT, at least in part, by inhibiting the TGF-β1/Smad3/integrin β1 signaling pathway.

## INTRODUCTION

Renal fibrosis is a common feature of progressive chronic kidney diseases and leads to compromised kidney functions or even end-stage renal failure [1]. Despite numerous intense studies, effective therapeutic avenues toward its treatment remain unavailable to date [2]. Epithelial–mesenchymal transition (EMT), a process wherein fully differentiated epithelial cells undergo transition to a mesenchymal phenotype, occurs in human fibrotic kidney and correlates with disease progression [3, 4]. Although contribution of EMT to the progression of renal fibrosis has been challenged by several lineage-tracing studies in mice [5], recently increasing evidence found that partial EMT program leads to dedifferentiation of renal epithelial cells and promotes kidney fibrosis [6, 7]. Thus, controlling EMT development may present an attractive perspective in preventing the progression of renal fibrosis [8, 9]. Unilateral ureteral obstruction (UUO) is a classic experimental model of renal tubular stress that generates progressive kidney fibrosis. EMT has been extensively found in kidney fibrosis following UUO [10, 11].

Transforming growth factor-β1 (TGF-β1) signaling is a major mediator in renal fibrosis and in EMT. The conventional TGF-β1 signals are transduced through transmembrane type I and II TGF receptors, which recruit phosphorylated Smad2 and Smad3 [12]. Subsequently, p-Smad2 and p-Smad3 heteroligomerize with the Smad4 and form an oligomeric complex that translocates into the nucleus. Then this complex activates or inactivates the transcription of target genes. Smad3 is a key mediator in TGF-β-induced fibrosis and EMT [13]. Inhibiting Smad3 activation and nuclear translocation blocks EMT [14] and renal fibrosis [10].

Cryptotanshinone (CTS, C_19_H_20_O_3_, molecular weight = 296.36) is a bioactive constituent isolated from the Chinese herb *Salvia miltiorrhiza*. CTS exhibits multiple pharmacological benefits, including anti-cancer [15, 16], anti-oxidative stress [17], and anti-cardiac fibrosis [18, 19]. CTS can evaluate MMP-2 [18] and decrease extracellular signal-regulated kinases 1/2 [19] to attenuate cardiac fibrosis. Moreover, CTS inhibits fibrosis to improve cardiac function by suppressing the STAT3 pathway in type 1-like diabetic rats model [20]. Additionally, CTS has the ability to decrease HS-derived fibroblastic (HSFs) cells producing collagen-1, collagen-3 and α-smooth muscle actin (α-SMA), so as to promote wound healing and decrease excessive deposition of extracellular matrix components [21]. However, the protective effects of CTS on kidney fibrosis have yet to be clarified. The present study aims to investigate the anti-fibrotic effects of CTS on kidney fibrosis and its possible mechanisms.

## RESULTS

### CTS prevents the progression of kidney injury and fibrosis in the UUO model

**Figure 1:**
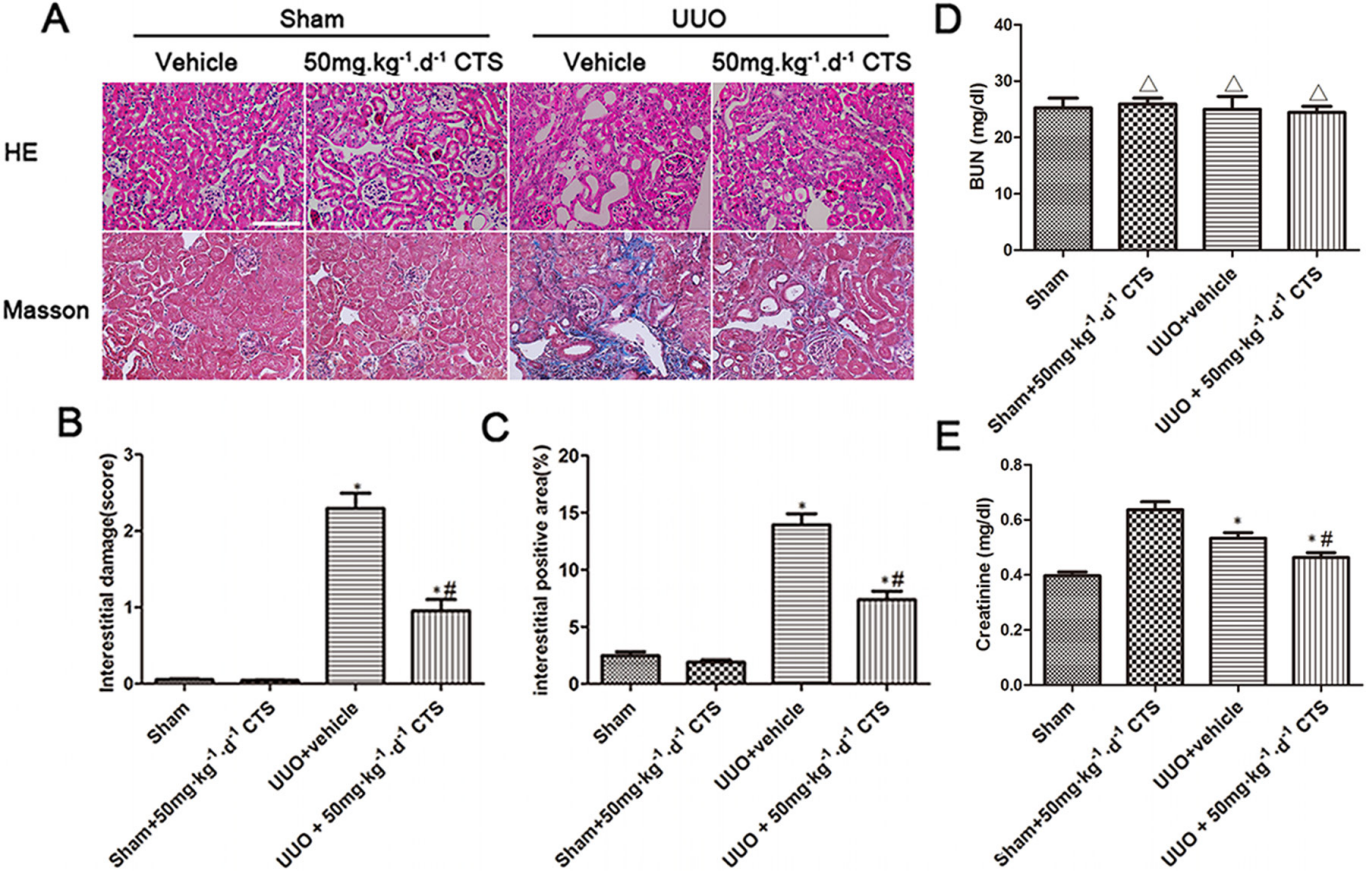
CTS alleviates renal injury and fibrosis in the seven-day UUO mouse model (**A**) Representative photomicrographs of H&E and Masson's trichrome staining, original magnification ×200; (**B**) Quantitative analysis for tubular injury score in H&E staining; (**C**) Quantitative analysis for Masson staining, blue area indicates collagen fibers; (**D** and **E**) Blood urea nitrogen (BUN) and Serum creatinine (Scr) of obstructed and non-obstructed kidneys. ^*^P < 0.05 vs. sham group, ^#^*P* < 0.05 vs. UUO + vehicle group. Scale bar, 200 μm. ^Δ^*P* > 0.05 vs. Sham group.

**Figure 2:**
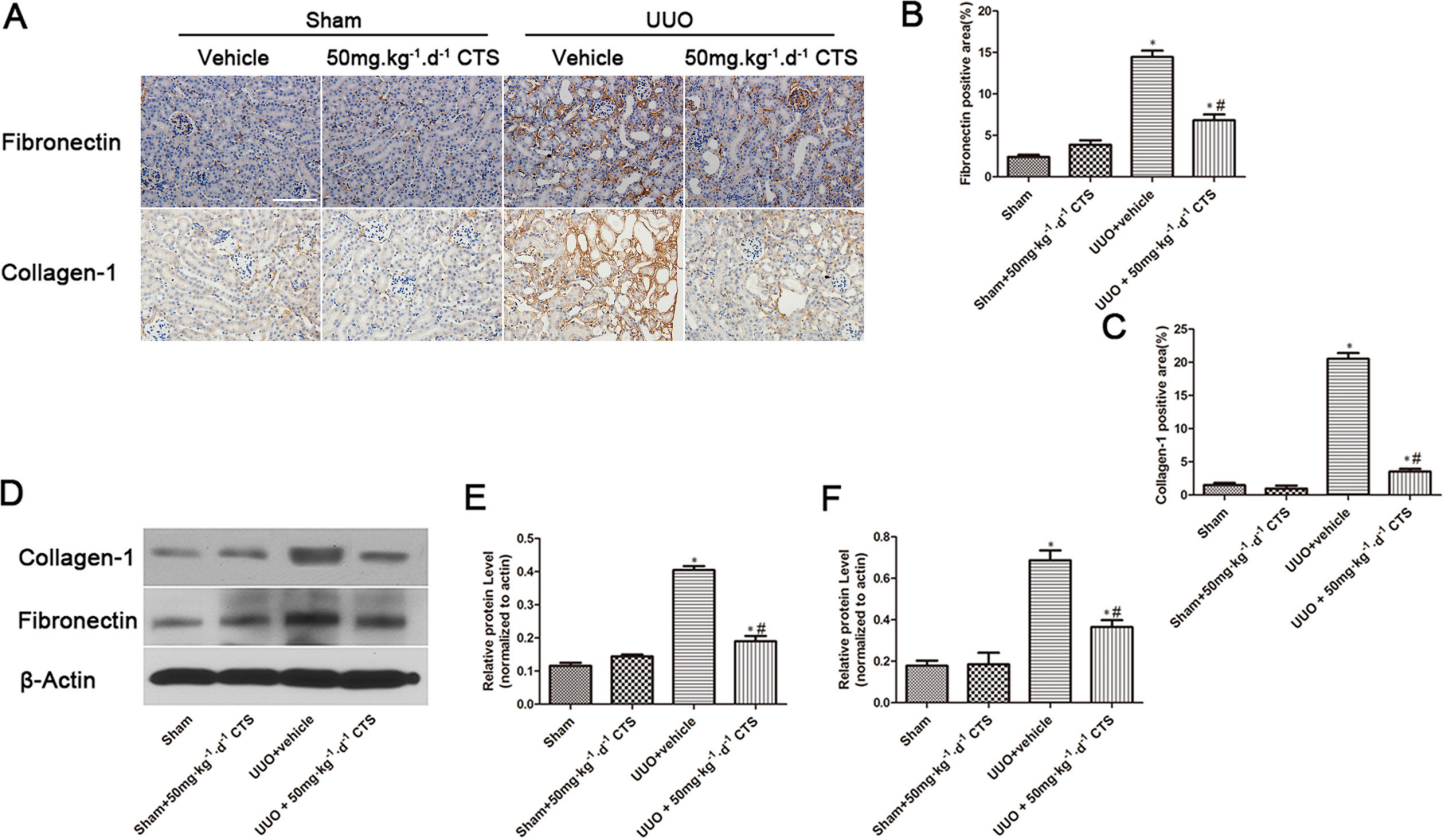
CTS decreases the deposition of ECM in the seven-day UUO mouse model (**A**) Immunohistochemical images for the expression of fibronectin and collagen-1, original magnification ×200; (**B** and **C**) Semiquantitative analysis for the IHC staining of fibronectin and collagen-1; (**D**) Representative images of Western blot gel for collagen-1 and fibronectin; (**E** and **F**) Semiquantitative analysis for Western blot of collagen-1 and fibronectin; ^*^P < 0.05 vs. sham group, ^#^*P* < 0.05 vs. UUO + vehicle group. Scale bar, 200 μm.

To evaluate whether CTS attenuates renal injury and fibrogenesis after UUO, histopathological analyses of fibrotic kidneys following H&E and Masson trichrome staining were performed after the treatment. H&E staining revealed significantly improved tubular expansion, epithelial atrophy, and infiltrated inflammatory cells after treatment with 50 mg⋅kg^-1^⋅day^-1^ CTS in the UUO model (Figure 1A). The 50 mg⋅kg-1⋅day-1 CTS-treated group showed a lower tubular injury score (0.96 ± 0.25) than the UUO + vehicle-treated mice group (2.30 ± 0.35) (Figure 1B) (*P* < 0.05). Masson trichrome staining showed a lower degree of interstitial collagen deposition in the 50 mg⋅kg^-1^⋅day^-1^ CTS-treated group than in the vehicle-treated group (Figure 1A and 1C) on day 7 after UUO. CTS had no effect on blood urea nitrogen value, but it markedly reduced the serum creatinine level after 7 days of UUO, which further conformed that CTS improved renal function to some extent (Figure 1D and 1E). Results of immunohistochemical staining and Western blot showed that the levels of interstitial matrix collagen-1 and fibronectin were lower in the 50 mg⋅kg^-1^⋅day^-1^ CTS-treated group than in the vehicle-treated UUO mice kidneys (Figure 2A–2F).

### CTS inhibits TGF-β1-induced ECM accumulation *in vitro*

**Figure 3:**
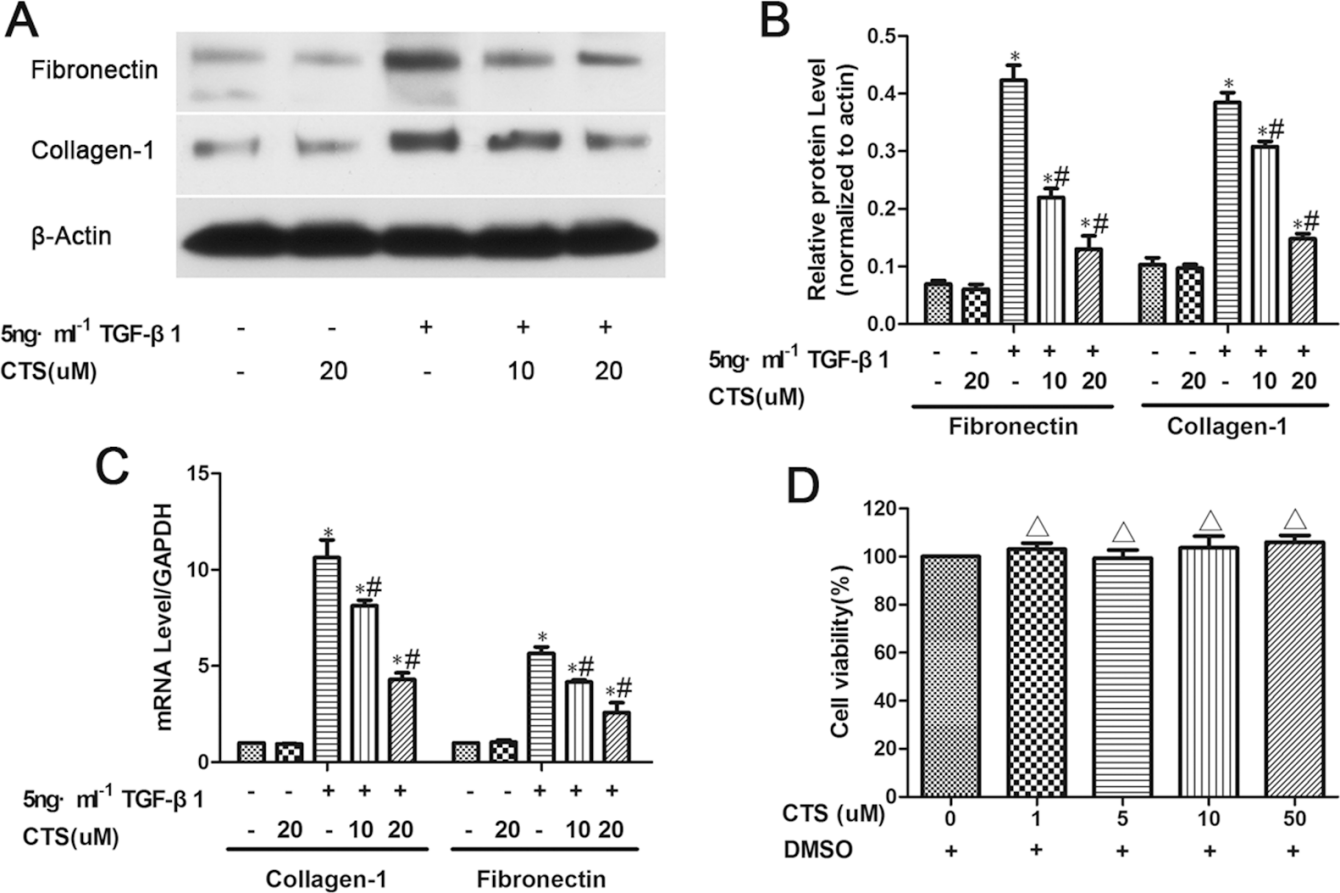
CTS decreases fibronectin and collagen-1 production in TGF-β1-treated NRK52E cells for 24 h (**A**) Representative images of Western blot for fibronectin and collagen-1. (**B**) Semiquantitative analysis of Western blot for fibronectin and collagen-1. (**C**) Real-time PCR analysis of mRNA levels of fibronectin and collagen-1. (**D**) The CCK-8 method detects cell viability after CTS treatment for 24 h. ^*^P < 0.05 vs. control, ^#^*P* < 0.05 vs. the former group. ^Δ^*P* > 0.05 vs. the control.

Considering that TGF-β1 is the most effective inducer of tubular fibrosis and EMT, we investigated the effects of CTS on TGF-β1-stimulated NRK52E cells for 24 h *in vitro*. The results of Western blot indicated that fibronectin and collagen-1 were overexpressed upon TGF-β1 treatment, but these effects were alleviated after treatment with 10 and 20 μM CTS (Figure 3A and 3B). Reverse transcription-PCR also demonstrated that collagen-1 and fibronectin were significantly increased in NRK52E cells after TGF-β1 stimulation, but this effect was abolished by CTS administration (Figure 3C). In addition, we tested the cytotoxic concentration of CTS on normal kidney NRK52E cells *in vitro* by using the CCK-8 method. CTS did not affect the viability rate of the cells under the pharmacological dose of 50 μM (Figure 3D).

### CTS hinders EMT in obstructed kidneys

**Figure 4:**
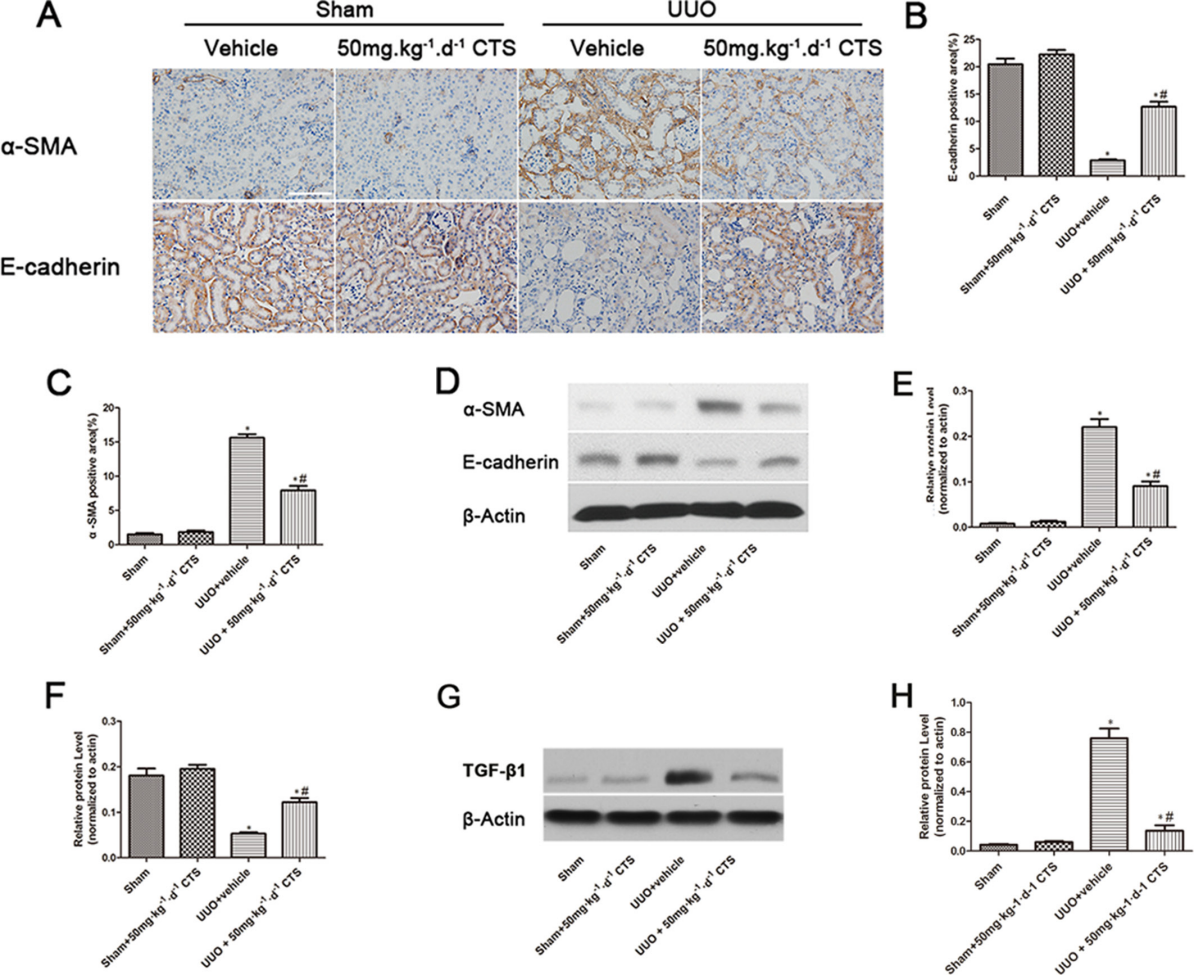
CTS blocks EMT in the seven-day UUO mouse model (**A**) Immunohistochemical images for the expression of E-cadherin and α-SMA, original magnification ×200; (**B** and **C**) Semiquantitative analysis for IHC staining of E-cadherin and α-SMA; (**D**) Representative images of Western blot for α-SMA and E-cadherin; (**E** and **F**) Semiquantitative analysis for Western blot of α-SMA and E-cadherin; (**G**) Representative images of Western blot for TGF-β1; (**H**) Semiquantitative analysis for Western blot of TGF-β1; ^*^P < 0.05 vs. sham group, ^#^*P* < 0.05 vs. UUO plus vehicle group. Scale bar, 200 μm.

Enrichment in transcription factors at the start of EMT correlates with kidney fibrosis progression. Therefore, we determined whether CTS could block this process to alleviate renal fibrosis. The results of immunohistochemical staining and Western blot for α-SMA and E-cadherin indicated that renal tubular epithelial cells lost their characteristic E-cadherin and acquired positive α-SMA expression, which was the mesenchymal cell's hallmark, after 7 days of UUO (Figure 4A–4F). However, intervention of CTS markedly reversed this process, as evidenced by α-SMA downregulation and E-cadherin retention. In addition, TGF-β1 was activated after 7 days of UUO, but this effect was decreased by CTS administration (Figure 4G–4H).

### CTS blocks TGF-β1 induced EMT for NRK52E cells *in vitro*

**Figure 5:**
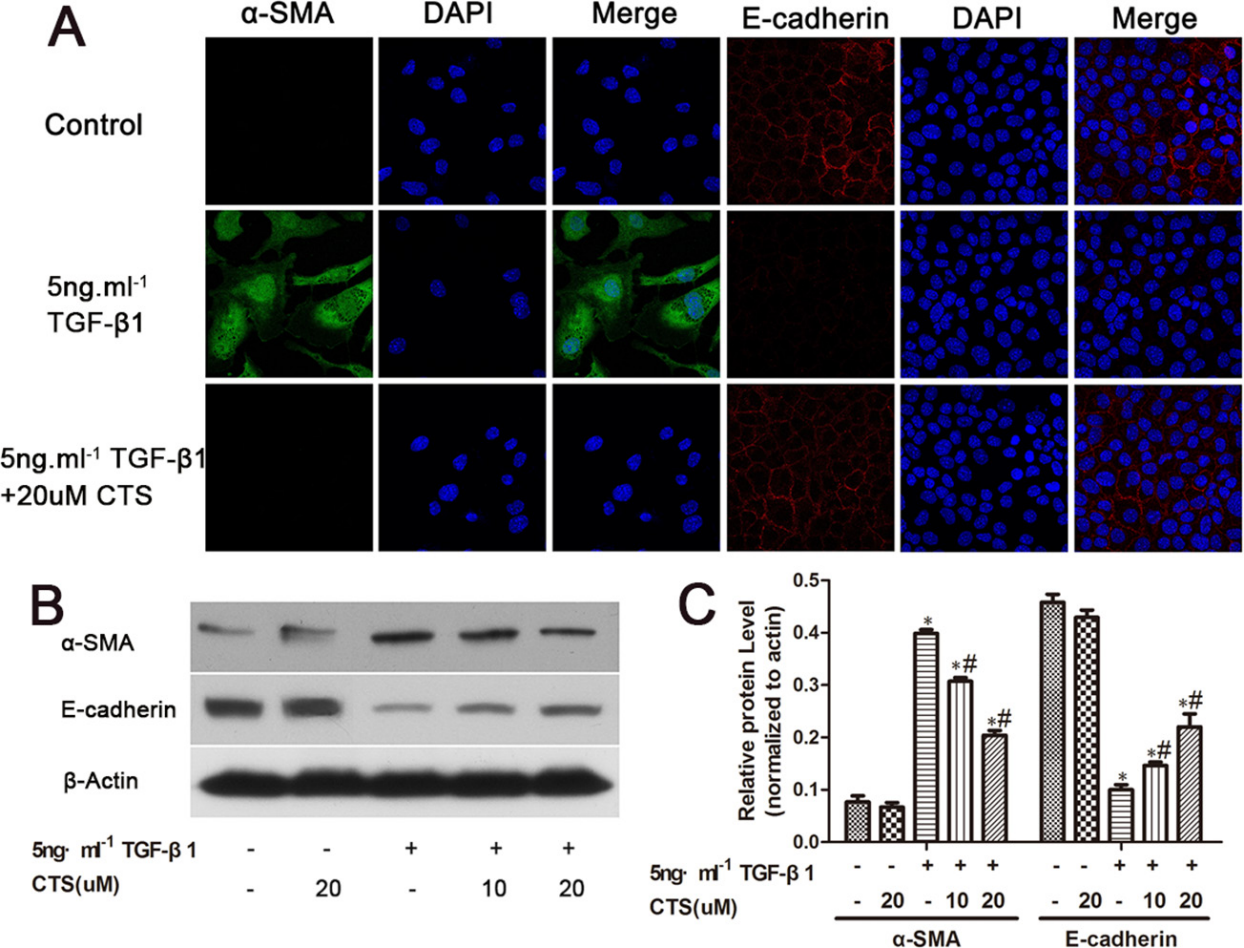
CTS decreases α-SMA but retains E-cadherin in TGF-β1-treated NRK52E cells for 24 h (**A**) Representative images of immunofluorescence staining for α-SMA and E-cadherin. Original magnification ×630. (**B**) Representative images of Western blot for α-SMA and E-cadherin. (**C**) Semiquantitative analysis of Western blot for α-SMA and E-cadherin. ^*^P < 0.05 vs. control, ^#^*P* < 0.05 vs. the former group.

Upon rhTGF-β1 treatment for 24 h, normal tubular NRK52E cells underwent phenotype transition, as proven by the immunofluorescent staining showing the absence of E-cadherin and de novo expression of α-SMA (Figure 5A). Similar to our *in vivo* data, CTS decreased the protein expression of α-SMA but retained that of E-cadherin (Figure 5B and 5C) in the TGF-β1-stimulated NRK52E cell model in a dose-dependent manner.

### CTS abolishes the phosphorylation of Smad3 but not MAPK signal

**Figure 6:**
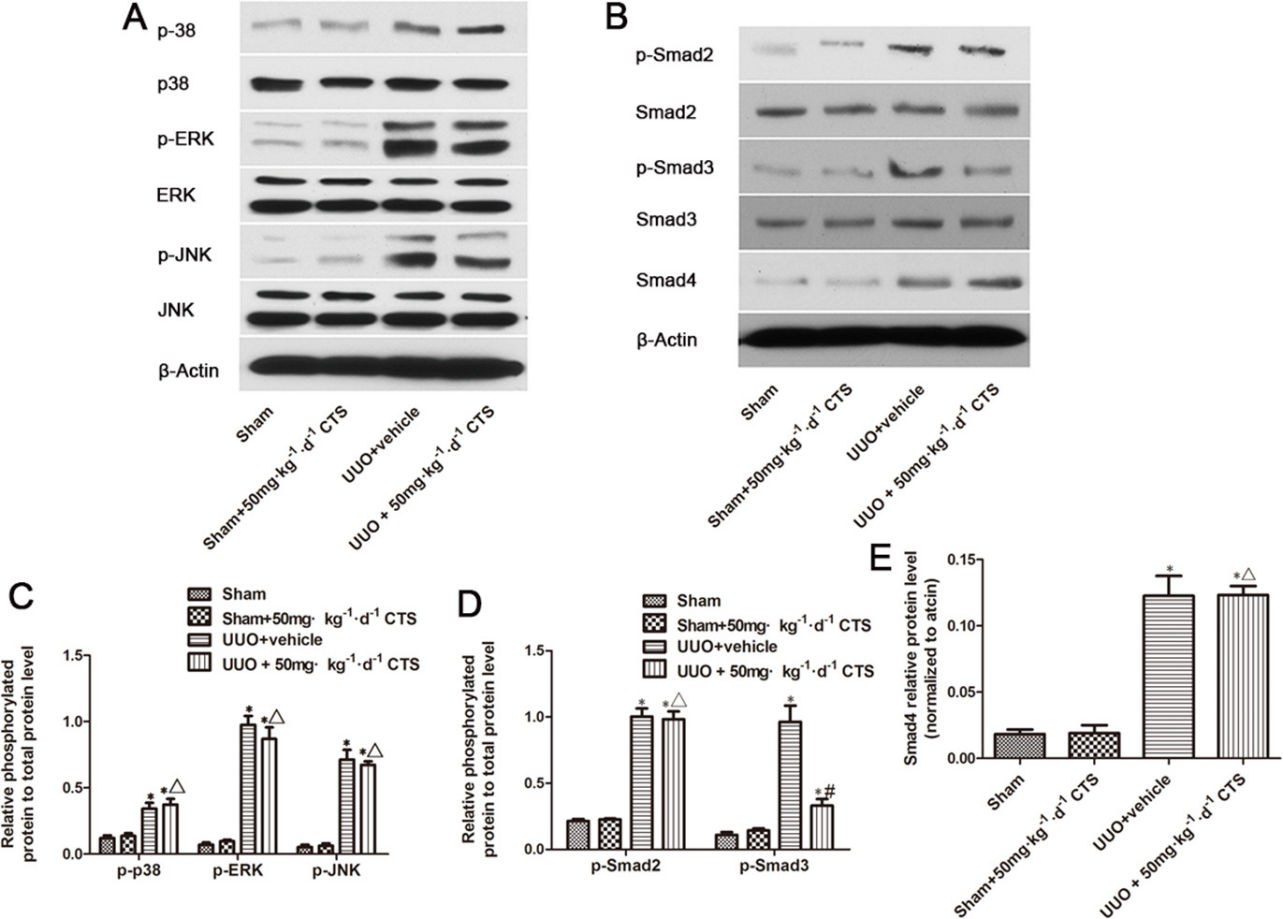
Cryptotanshinone inhibits the activation of Smad3 in the seven-day UUO mouse model (**A**) Representative images of Western blot for p-p38, p38, p-ERK, ERK, p-JNK, and JNK. (**B**) Representative images of p-Smad2, Smad2, p-Smad3, Smad3, and Smad4. (**C** and **D**) Semiquantitative analysis for Western blot of phosphorylated protein level to total protein level. (**E**) Semiquantitative analysis for Western blot of Smad4. ^*^P < 0.05 vs. sham group, ^#^*P* < 0.05 vs. UUO plus vehicle group. ^Δ^*P* > 0.05 vs. UUO plus vehicle group.

TGF-β1 signaling activates EMT. Smads proteins and non-Smad pathway P38 mitogen-activated protein kinase (MAPK) are major downstream signaling mechanisms implicated in renal fibrosis and EMT. In this study, we examined whether CTS could inhibit these two signals to block EMT. Both signals were activated after 7 days of UUO, as evidenced by increased p-P38, p-ERK, p-JNK, p-Smad2, and p-smad3. Treatment with CTS only inhibited the phosphorylation of Smad3 but not that of Smad2 or Smad4 (Figure 6). In addition, CTS did not alter the phosphorylation of p38, ERK, and JNK in the UUO model.

### CTS selectively inhibits smad3 activation and transcription *in vitro*

**Figure 7:**
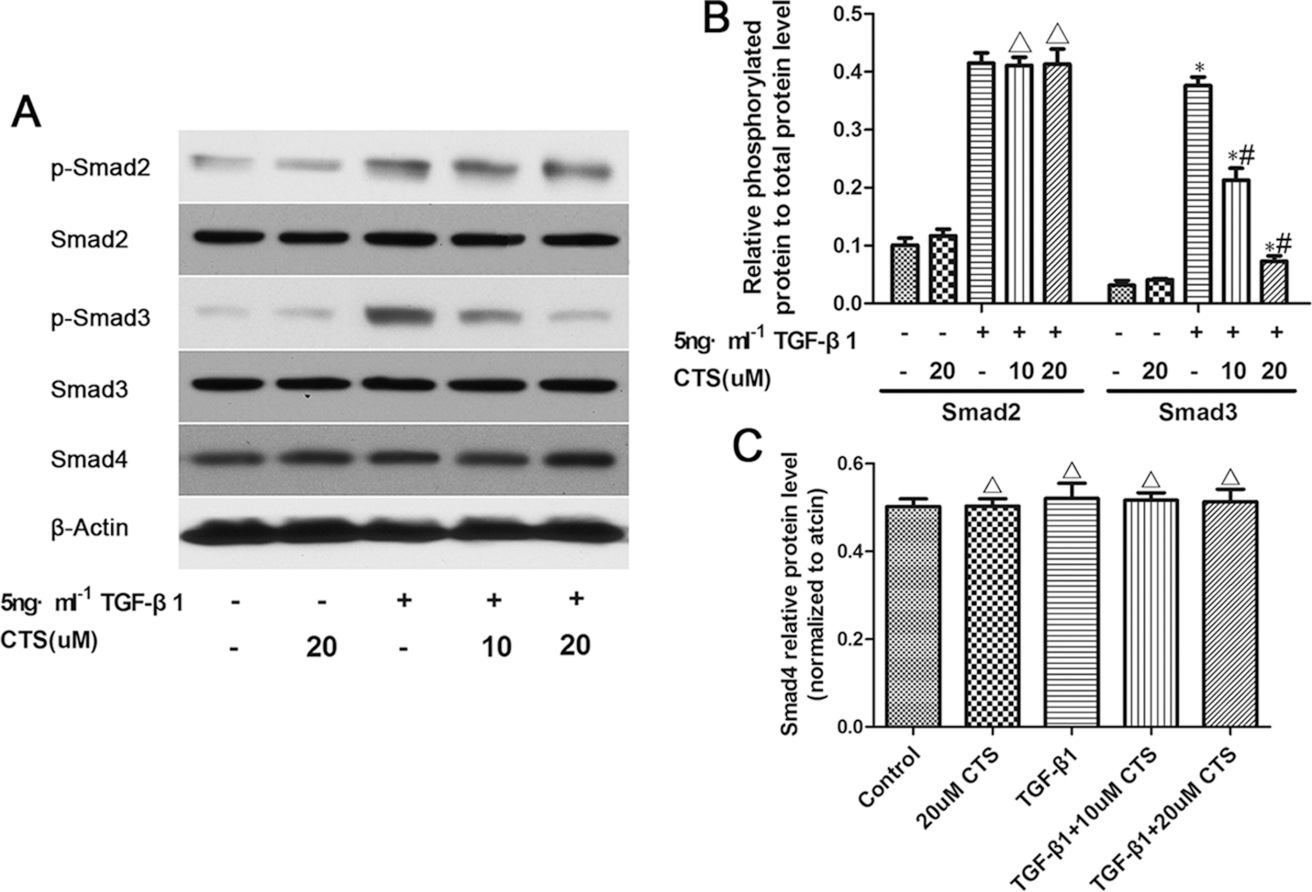
CTS selectively inhibits the phosphorylation of Smad3 but exhibits no effect on that of Smad2 or Smad4 (**A**) Representative images of Western blot for p-Smad2, Smad2, p-Smad3, Smad3, and Smad4. (**B**) Semiquantitative analysis for Western blot of phosphorylated protein level to total protein level. (**C**) Semiquantitative analysis of Western blot for Smad4. ^*^P < 0.05 vs. control, ^#^*P* < 0.05 vs. the former group. ^Δ^*P* > 0.05 vs. the former group.

**Figure 8:**
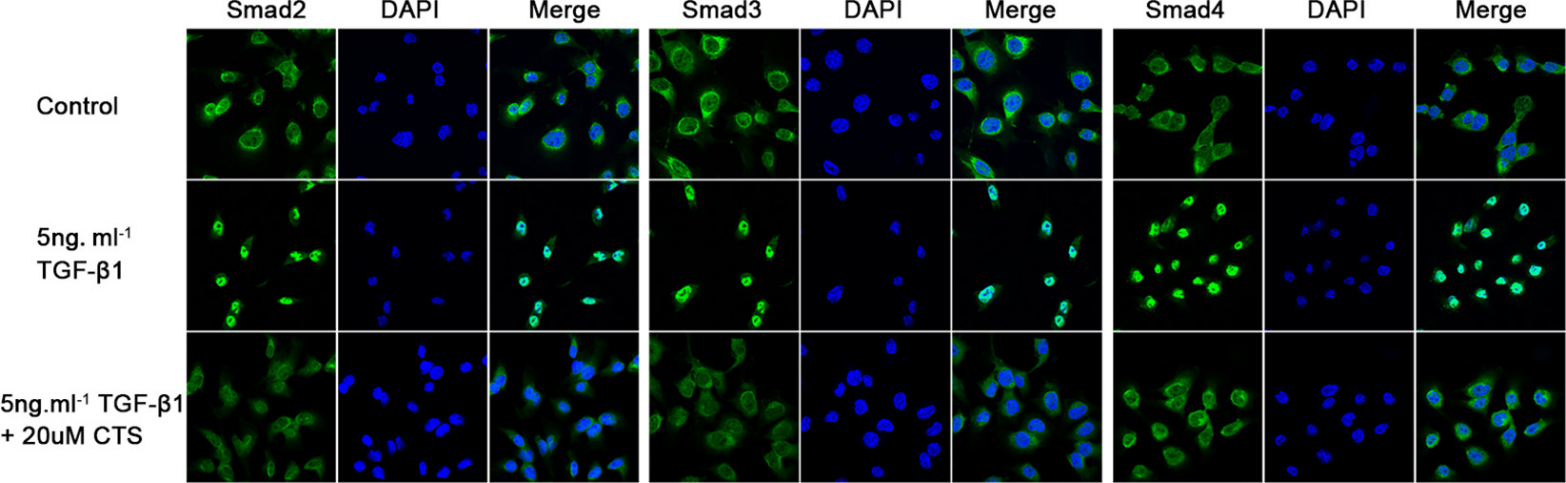
CTS inhibits Smads complex nuclear translocation Representative images of immunofluorescence staining for Smad2, Smad3, and Smad4.

Considering that CTS can block EMT *in vivo* and *in vitro*, we investigated the possible mechanism underlying this phenomenon. One possible mechanism involves CTS selectively suppressing the phosphorylation of Smad3 *in vitro*. To verify our assumption, TGF-β1-stimulated NRK52E cells were co-cultured with two concentrations of CTS (10 and 20 μM) for 1 h. TGF-β1 treatment dramatically increased p-Smad2 and p-Smad3 but did not influence Smad4. Exposure to CTS at 10 and 20 μM reduced the phosphorylation of smad3 by 50% and 75%, respectively. However, no significant change in p-Smad2 or Smad4 level was observed (Figure 7). Treatment with 20 μM CTS also markedly inhibited the TGF-β1-induced nuclear translocation of Smad3, Smad2 and Smad4, as demonstrated by the results of immunofluorescence staining in Figure 8.

### CTS suppresses Smad3-induced integrin β1 promoter activity

**Figure 9:**
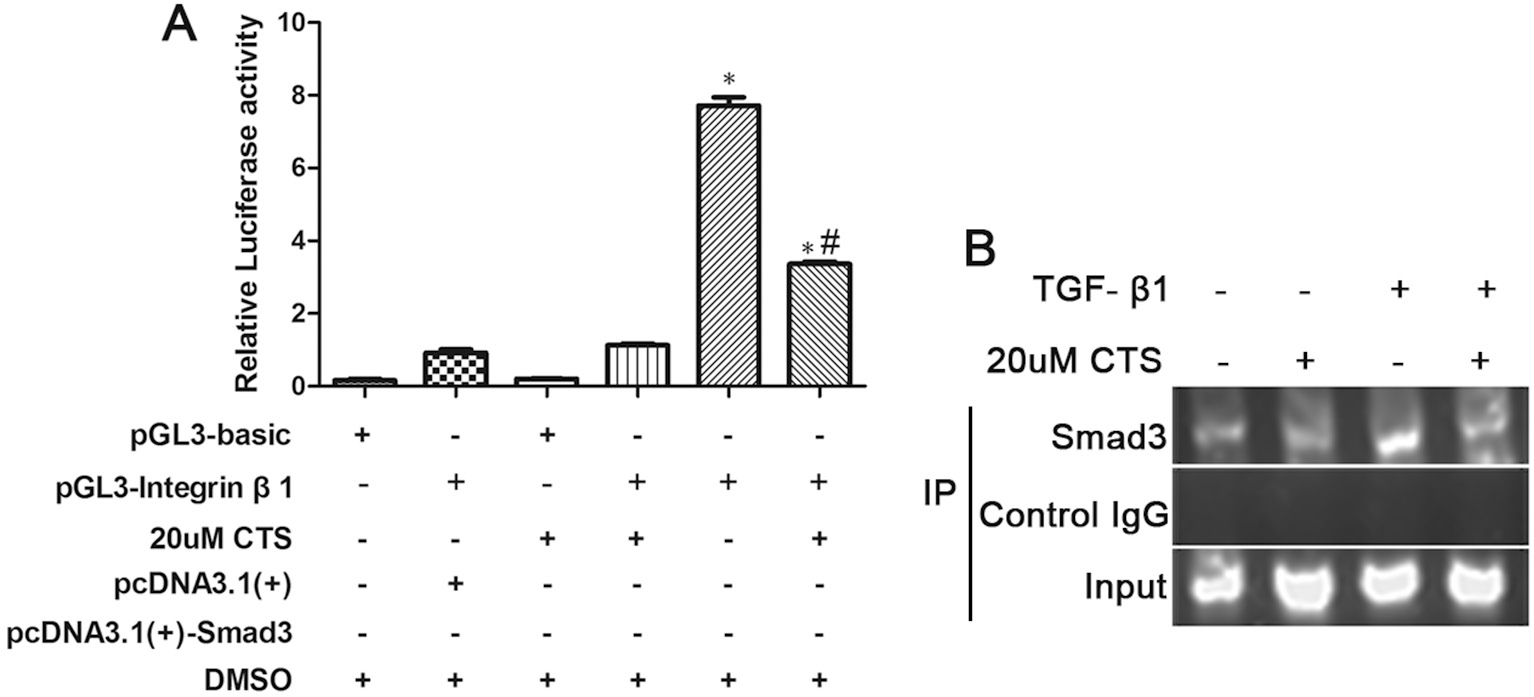
CTS suppressed integrin β1 promoter activity through Smad3 (**A**) Luciferase assay of integrin β1 promoter activity was measured 24 h after transfection. (**B**) Chromatin immunoprecipitation analysis for Smad3 binding to the core region sequence on integrin β1 promoter. After treating NRK52E cells with or without TGF-β1 for 12 h, PCR was performed to determine the core binding element on integrin β1 promoter. ^*^P < 0.05 vs. PGL3-integrin β1 plus pc DNA 3.1 (+) group; ^#^*P* < 0.05 vs. the former group.

Integrin β1 has been reported activated in TGF-β1-induced EMT. Blockade of integrin β1 signaling impaired TGF-β1 mediated EMT and subsequent kidney fibrosis. Smad-binding element (SBE) is present on integrin β1 promoter sites. Smad3 could bind to the integrin β1 promoter in mouse cells [22]. Then, we conducted luciferase activity assay to determine whether CTS could inhibit the integrin β1 promoter activity activated by Smad3. As shown in Figure 9A, the cells transfected with pGL3-integrin β1 or pcDNA3.1(+) only showed basal luciferase activities, whereas it was significantly increased after co-transfection with pGL3-integrin β1 and pcDNA3.1(+)-Smad3. Integrin β1 promoter activity was markedly suppressed after treatment with 20 μM CTS for 24 h. In addition, the results of chromatin immunoprecipitation indicated that 12 h treatment of TGF-β1 increased the Smad3 binding to the SBE region on integrin β1 promoter, but this direct interaction was decreased after treatment with 20 μM CTS (Figure 9B).

## DISCUSSION

Deposition of ECM in renal interstitium is a main feature in the pathogenesis of progressive renal fibrosis [23]. ECM is composed of collagen types I and IV, fibronectin, and vimentin, which are mainly produced by myofibroblasts, but its origin remains controversial [24]. Nowadays, accumulating evidence demonstrated myofibroblasts arising from a diverse origins: activated renal fibroblasts, endothelial-to-mesenchymal transition (EndoMT), EMT, pericytes, fibrocytes, bone marrow derived cells and bone marrow derived cells [23]. The activated epithelial cells lose their adhesion proteins and acquire mesenchymal characters such as α-SMA and fibroblast surface protein (FSP-1). Then, the tubular basement membrane is disrupted, the cells migrate, and their invasion ability enhances [25]. Renal tubular epithelial cells in injured kidneys undergo a phenotypic transformation into matrix-producing myofibroblasts by EMT. Even though a large number of studies have suggested the possibility of EMT in kidney fibrosis *in vivo*, it still challenged by some studies [26, 27]. EMT was first observed by Strutz et al. in 1995, who noted that Fsp1-positive fibroblasts originate from the local conversion of the epithelium in a mouse model of anti-tubular basement membrane disease [28]. Later, EMT was largely reported in various animal models of kidney fibrosis, including diabetic nephropathy [29], obstructive nephropathy [30], and 5/6 nephrectomy [31] models. These pieces of evidence demonstrated that EMT was a crucial event during renal fibrogenesis, and it was targeted to suppress renal fibrosis [32–34]. Our *in vivo* and *in vitro* data showed that CTS elicits a protective effect on renal tubulointerstitial fibrosis. In addition, EMT was activated in the progression of renal fibrosis, and CTS treatment decreased the α-SMA-positive epithelial cells and sustained their E-cadherin expression levels. In thus Thus, CTS could block EMT. In addition, the results of the CCK-8 method indicated that CTS exerted no cytotoxic effects on normal renal tubular epithelial cells, which could be a useful motif in clinical applications.

TGF-β1 is a principal effector cytokine responsible for the induction of kidney fibrosis and EMT. It acts mainly through a canonical pathway that activates Smad2 and Smad3 or through non-canonical signaling pathways to induce EMT in fibrotic kidneys [35]. Then, TGF-β1/Smads and MAPK signals were measured to understand the molecular mechanism underlying the protective effect of CTS toward EMT. CTS did not affect the protein expression levels of MAPK in the UUO model, but it selectively inhibited the phosphorylation of Smad3 and not that of Smad2 or Smad4. In addition, our *in vitro* data confirmed these results. CTS markedly blocked the TGF-β1-induced nuclear translocation of Smad2, Smad3 and Smad4, which suggested the phosphorylation of Smad3 required by the nuclear translocation of Smad2 and Smad4. Smad3 promotes EMT by directly binding to the promoter regions of targeted genes [13], but Smad2 cannot directly bind to the genomic sequences [36]. Consequently, we wanted to explore CTS-targeted downstream proteins of Smad3.

Integrin β1 is a molecular mediated ECM signaling that activates many growth factors, including hepatocyte growth factor and epidermal growth factor. Blocking integrin β1 downstream signals such as Integrin-linked protein and Focal Adhesion Kinase significantly inhibits TGF-β1-induced EMT [37, 38]. A previous study reported that the TGF-β1-induced activation of integrin β1 was Smad3 dependent [22]. Smad3 could bind to the promoter region of integrin β1 and then mediated EMT and subsequent renal fibrosis. Data from luciferase assay showed that CTS suppressed the integrin β1 promoter activity increased by Smad3. Chromatin immunoprecipitation assay further indicated that CTS conld block the direct interactions of Smad3 and SBE on integrin β1 promoter. This phenomenon could be a potential consequence of the inhibitory effect of CTS on Smad3 phosphorylation.

Overall, our study indetifies CTS as an anti-fibrotic agent that attenuates renal ECM accumulation and prevents the initiation of EMT *in vitro* and *in vivo*. The possible mechanism comprises the inhibition of TGF-β1/Smad3/integrin β1 signaling pathway, and well-designed prospective clinical trials are needed before its use can be warranted in clinical practice.

## MATERIALS AND METHODS

### Chemicals and antibodies

Antibodies to collagen-1, fibronectin, and α-SMA were purchased from Abcam (Cambridge, MA, USA). p-Smad3, Smad3, p-Smad2, Smad2, p-JNK, JNK, p-p38, p38, p-ERK1/2, TGF-β1 and ERK1/2 were obtained from Cell Signaling Technology (Dancers, MA, USA). Antibody to E-cadherin was procured from BD Biosciences (San Diego, CA, USA), and Recombinant Human TGF-β1 was purchased from Millipore (Billerica, MA, USA). CTS was purchased from Xi’an Hong-sheng Bio-Tech Co., Ltd. (Xi’an, Shanxi, China). CTS was dissolved in dimethyl sulfoxide (DMSO) at various times. The final concentration of DMSO in the media was less than 0.1%.

### Unilateral ureteral obstruction (UUO) model

Forty wild-type C57/BL6 mice aged 8–10 weeks (20–25g) were obtained from the Beijing HFK Bioscience Co., Ltd. (Beijing, China). All the procedures performed for animal housing and surgeries in our studies complied with the guidelines of the Institutional Animal Care and Use Committee at Wuhan University, China. All experimental protocols were approved by the Animal Experimentation Ethics Committee at Wuhan University. All mice were randomly divided into four groups (*n* = 10): sham operation + vehicle; sham operation + 50 mg⋅kg-1⋅day-1 CTS; UUO + vehicle (0.1% DMSO); and UUO + 50 mg⋅kg^-1^⋅day^-1^ CTS. CTS was dissolved in 0.1% DMSO (Sigma–Aldrich Company Ltd., Dorset, UK) and used for gastric gavage administration from 7 days before UUO operation to one week after surgery. The dosage of CTS was derived from a previous study and tested based on our pilot test to confirm whether the pathology staining in the UUO model was improved [14]. UUO surgery was performed as previously described [39]. Seven days after surgery, the mice were euthanized and the kidneys were harvested. Meanwhile, serum samples were collected to detect the levels of blood urea nitrogen and serum creatinine using an AU5800 automatic biochemistry analyzers (Beckman Coulter Inc., Kraemer Boulevard Brea, CA, USA). Half of the kidney was fixed in 10% phosphate-buffered formalin and then embedded in paraffin for histological studies. The remaining kidneys were snap-frozen in liquid nitrogen and then stored at −80 °C for protein and RNA extractions.

### Western blot analysis

Kidney tissues were treated as indicated and homogenized in ice-cold-modified RIPA lysis buffer (Biyuntian, Haimen, China) with a polytron homogenizer (IKA GmbH, Königswinter, Germany). The lysates were collected after centrifuged at a speed of 12,000 ×g at 4°C for 20 min. The proteins (20–50 μg) were mixed with SDS and then denatured using a heating set. The supernatants were loaded on an 8%–12% polyacrylamide gel and then separated through SDS–polyacrylamide gel electrophoresis. The proteins were transferred onto polyvinylidene fluoride membranes (Millipore, Billerica, MA, USA) at 4°C for 2 h. The membranes were blocked in 5% non-fat milk diluted using 0.02% Tris Buffered Saline with Tween-20 (TBST) at room temperature for 1 h before being probed with various primary antibodies overnight at 4 °C in a blocking buffer. The blots were then probed with respective horseradish peroxidase-conjugated secondary antibodies (Santa Cruz Biotechnology, Dallas, Texas, USA) at room temperature for 1 h, and then washed three times with TBST. Finally, the bound secondary antibodies were detected using the enhanced chemiluminescence kit (Thermo Fisher Scientific, Waltham, MA, USA). β-actin was loaded as an internal control.

### Cell culture and treatments

NRK52E cells were originally obtained from the American Type Culture Collection (Manassas, VA, USA) and maintained in Dulbecco's modified Eagle's medium-F12 medium (GIBCO, Grand Island, NY, USA). For TGF-β1 treatment, NRK52E cells were seeded onto six-well plates at 60% confluence and then cultured in complete medium overnight. After the cells suffered 24 h serum starvation, the cells were treated with 5 ng/mL recombinant human TGF-β1 in serum-free medium for the indicated time period. CTS was dissolved in DMSO and added to the medium 2 h before TGF-β1 treatment. The percentage survival of the cells was determined using the Cell Counting Kit-8 (CCK-8) method in accordance with the manufacturer's instructions (Beyotime, Haimen, China). The absorbance (A) was measured at 450 nm, and the cell survival rate was calculated using the following formula: Cell survival rate (%) = (A of the experimental group/A of the control group) × 100.

### Histological examination

Kidney tissue fixed in 4% paraformaldehyde was processed by dehydration and embedded in paraffin. Sections were cut at 4 μm as previously described. Masson trichrome or hematoxylin and eosin (H&E) staining was performed in accordance with the manufacturer's protocol (Sigma–Aldrich, St. Louis, MO). Kidney injuries were evaluated using Dr. Islam M's methods as previously described [40]. Fibrotic cortical interstitial areas were detected by Masson trichrome staining. The UltraVision Quanto Detection System HRP DAB kit (Thermo Fisher Scientific, Waltham, MA, USA) was used for the staining process in immunohistochemical analysis. The procedures were described briefly as follows. Tissue sections were deparaffinized in xylene and hydrated in graded ethanol before antigen retrieval. Endogenous peroxidase activity was blocked by 3% hydrogen peroxide. After blocking nonspecific binding sites with blocking buffer, the sections were incubated with primary antibodies overnight at 4°C. Then, the tissues were incubated with corresponding secondary antibodies (Santa Cruz Biotechnology, Dallas, Texas, USA) at room temperature for another 1 h. Diaminobenzidine was used to detect the proteins. We randomly selected 10 fields to photograph images under a microscope at 200× magnification (Olympus, Japan) and measure positive signals using Image Pro-Plus 6.0 software.

### Immunofluorescence staining

Indirect immunofluorescence staining was performed on various treated NRK52E cells on coverslips. In brief, the cells cultured on coverslips were washed with PBS, fixed in 4% paraformaldehyde for 20 min, and then permeabilized with 0.4% Triton X-100 (except for E-cadherin). After washing three times extensively with PBS, the slides were blocked with 1% bovine serum albumin (BSA, Sigma) and then exposed to the primary antibodies overnight at 4°C. Afterward, the slides were incubated with secondary antibodies conjugated with fluorescein isothiocyanate (Invitrogen, Carlsbad, CA, USA). Then, the nuclei were counterstained with Hoechst 33342 (Invitrogen, Carlsbad, CA, USA). Images were captured through confocal microscopy (Olympus Corporation, Tokyo, Japan).

### Real-time PCR

RNA was extracted from the kidney cortex by using Trizol reagent (Invitrogen, Carlsbad, CA, USA) in accordance with the manufacturer's protocol. Reverse transcription-PCR was conducted using the PrimeScriptTM RT reagent kit (Takara, Japan), and glyceraldehyde 3-phosphate dehydrogenase (GAPDH) was used as an internal control. All reactions were conducted in a 25 μL volume. Real-time reactions were performed with SYBR Premix Ex Taq II (Takara, Japan) by using the AB7500 Real-time PCR system (Thermo Fisher Scientific, Waltham, MA, USA). The primer sequences were given as follows: 5′-AACGGCAAGGTGTTGTGCGATG′ (forward), 5′-AGC TGGGGAGCAAAGTTTCCTC-3′ (reverse) for collagen-1; 5′-ACTGGCGTCCCACGATCCGA-3′ (forward), 5′-GGA GCGGGGGTCCAGGTGAT-3′ (reverse) for fibronectin; and 5′-CAAGGTCATCCATGACAACTTTG-3′ (forward), 5′-GTCCACCACCCTGTTGC TGTAG′ (reverse) for GAPDH. PCR was performed under the following conditions: initial denaturation at 95°C for 30 s and then 40 cycles of 5 s at 95°C, 30 s at 60°C, and 1 min at 72°C. The threshold cycle values of each sample were measured using the 2^−ΔΔCT^ data analysis method.

### Plasmid construction and luciferase reporter assays

The rat Smad3 cDNA was amplified using PCR and inserted into pcDNA 3.1 (+) at HindIII and EcoRI restriction enzyme cutting sites. The primer sequences were Smad3-F: 5′CCCAAGCTTATGTCGTCCATCCTGCCCTTC3′ (HindIII), Smad3-R: 5′CCGGAATTCCTAAGACACGC TGGAACAGCG3′ (EcoRI). The promoter regions of integrin β1 (−940–+170) were amplified using PCR and cloned into pGL3-basic plasmid at Nhel and BgIII restriction enzyme cutting sites. The primer sequences were forward 5′-CTAGCTAGCTCACCGCTGGGCTGT-3′ Nhel, Reverse 5′-GGAAGATCTCTTTTCGCAGCGT CCGCC-3′ BgIII. The NRK52E cells were seeded onto six-well plates. Cells at 60% confluence were co-transfected with pcDNA 3.1 (+)-Smad3 and (or) pGL3-integrin β1 promoter. The pSV-β Gal plasmid (Promega, Madison, WI, USA) was used as an internal control to normalize the transfection efficiency. At 24 h after the transfection, the cells were washed with PBS and then lysed with passive lysis buffer (Promega, Madison, WI, USA). The luciferase activity in each group was measured using the Dual-Luciferase Reporter Assay System in accordance with the manufacturer's protocol (Promega, Madison, WI, USA). β-Galactosidase activities were measured using the luminescent β-galactosidase detection kit II (Clontech Laboratories, Inc., Mountain View, CA, USA).

### Chromatin immunoprecipitation

Cells were seeded onto six-well plates and treated with 5 ng/mL TGF-β1 or (and) 20 μM CTS. At 12 h after treatment, the cells were washed with cold PBS and then cross-linked in 1% formaldehyde at 37°C on a rocking or shaking device for 15 min. Cross-linking was terminated by adding 125 mM glycine. The cells were rinsed three times with PBS and then collected in PBS. After centrifugation at 1000 × g for 5 min, the cells were re-suspended with sonication buffer (containing protease inhibitor cocktail at a dilution of 1:1000) and then sonicated to shear DNA to lengths between 200 and 1500 bp. A 50 μL aliquot was taken as input, and DNA concentration was measured. Protein A/G beads were washed three times with sonication buffer and then pre-incubated with sonication buffer containing 1 g/mL sonicated sperm DNA + 1 mg/mL BSA for 4 h. Beads were added to the 10-fold diluted sonicated lysates and then rotated at 4°C for 2 h. After brief centrifugation, the Smad3 antibody (Santa Cruz, Dallas, Texas, USA) and the control rabbit IgG (Santa Cruz, Dallas, Texas, USA) antibody were added to the supernatant and then rotated at 4°C overnight. Then, the immune complexes were obtained by rotating with protein A/G beads at 4°C for 3 h. After gentle and subsequent centrifugation, the beads were washed with wash buffers and finally washed with TE buffer. The immune complexes were eluted from the beads with 1% SDS in TE, and the DNA–protein cross-links were reversed by treatment with NaCl and heating the samples at 65°C for 4 h. The products were purified using a PCR purification kit (Qiagen, Hilden, Germany) and detected by RT-PCR. The resultant immunoprecipitates were subjected to Nested PCR using specific primers for amplifying regions corresponding to the Smad-binding sequence on the integrin β1 promoter region. The outer primers for first-round PCR amplification was 5′-GGCTTCGGCTGGGCTTTC-3′ (forward), 5′-AGTGAGTGGGCGGAGACT-3′ (reverse); Tm = 62°C, for 20 cycles. First-round cDNA production was then diluted to a thousand percentage with TE buffer and then subjected to second-round PCR with the primer sequences 5′-GGCTTCGGCTGGGCTTTC-3′ (forward), 5′-AGGCGGCAGGAAGAAGGA-3′ (reverse); Tm = 62°C, for 30 cycles.

### Statistical analysis

The *in vitro* and *in vivo* experiments were performed in a double- or semi-blinded fashion, and the treatment protocols or groups were randomized. All experiments were repeated at least five times independently. Continuous data were expressed as mean ± SD. Difference between groups were statistically analyzed using one-way ANOVA followed by Student-Newman-Keuls’ method with SPSS 19.0 software. Statistical significance was considered at *P* < 0.05.

## Abbreviations

CTS: Cryptotanshinone
TGF-β1: Transforming growth factor-1
EMT: Epithelial–mesenchymal transition
UUO: Unilateral ureteric obstruction
MAPK: Mitogen-activated protein kinase
ECM: Extracellular Matrix
FSP-1: Fibroblast surface protein
ILK: Integrin-linked protein
FAK: Focal Adhesion Kinase
